# Postoperative care for paediatric cataract patients

**Published:** 2016

**Authors:** P Vijayalakshmi, Lucy Njambi

**Affiliations:** Chief: Paediatric Ophthalmology & Strabismus Department, Aravind Eye Hospital, Madurai, India.; Lecturer and paediatric ophthalmologist: University of Nairobi, Nairobi, Kenya.

**Figure:**
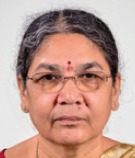
Dr P Vijayalakshmi

**Figure:**
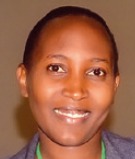
Lucy Njambi

Alongside good quality care before and during a cataract operation, careful postoperative care and long term follow-up are essential for good outcomes in children undergoing cataract surgery. This is only possible with the active and ongoing involvement of parents. By giving their child the medication prescribed for them, at the correct times, parents play a vital role in helping the eye to heal well and reducing complications. By bringing their child back for refraction and regular follow-up appointments, parents help to ensure a good visual outcome. Every effort should be made to support parents, for example:

- Even before the operation, discussing with them the important role they have to play.
- Giving them oral and written information regarding medication and follow-up visits before they leave the hospital.
- Putting in place a system or personnel to track patients and send reminders about medication compliance and appointments.

For the eye team, it is important to be aware of children's particular postoperative needs. Children's eyes are different from adults' eyes, and are more prone to severe inflammation (uveitis) and a shallow anterior chamber after cataract surgery. Accurate refraction is critical because of the risk of amblyopia, but correction is more complex in young children as their eyes continue to grow and their refractive status can change over time. The risk of opacity of the visual axis, and the risk of glaucoma, are also far greater in children than in adults. As with adult cataract surgery, endophthalmitis can also occur, but is rare.

**‘Personnel working in the recovery room must pay attention to the child as well as the operated eye’**

In this article, we look at the postoperative care required at various stages, and on page 34 we discuss complications, amblyopia, surgery on the second eye and insertion of a secondary IOL.

## Immediately after surgery, in the recovery room

Personnel working in the recovery room must pay attention to the child as well as the operated eye. An eye shield should be secured in place to protect the operated eye. Pain and vomiting must be controlled as both can raise the intraocular pressure, leading to a shallow anterior chamber and/or displacement of the IOL

After safe extubation, vital signs should be monitored for 60–90 minutes in a designated postoperative recovery room. The recovery room should have well trained staff and have a controlled temperature, with a well equipped system for oxygen administration, pulse oximetry, cardiac monitoring and suction. Fever and shivering after general anaesthesia are common. The child should be kept warm, using a warmer for very young infants, and should be placed on her or his side with the operated eye uppermost. Fever can be managed with paracetamol syrup. Oxygen saturation and pulse rate should be monitored and the child observed for signs of respiratory distress, nausea or vomiting. An anaesthetist or anaesthesia technician trained in the care of young children should always be available.

Once the child has completely recovered, she or he can be moved to the ward. Parents must be told how and when to feed the child and how to avoid choking (which can increase pressure in the eye).

## On the ward

On the ward, the child should be observed for restlessness, irritability or crying (which is usually due to pain). It is important to control pain as crying can also raise the intraocular pressure or the child may vigorously rub the eye.

**Figure 1.**
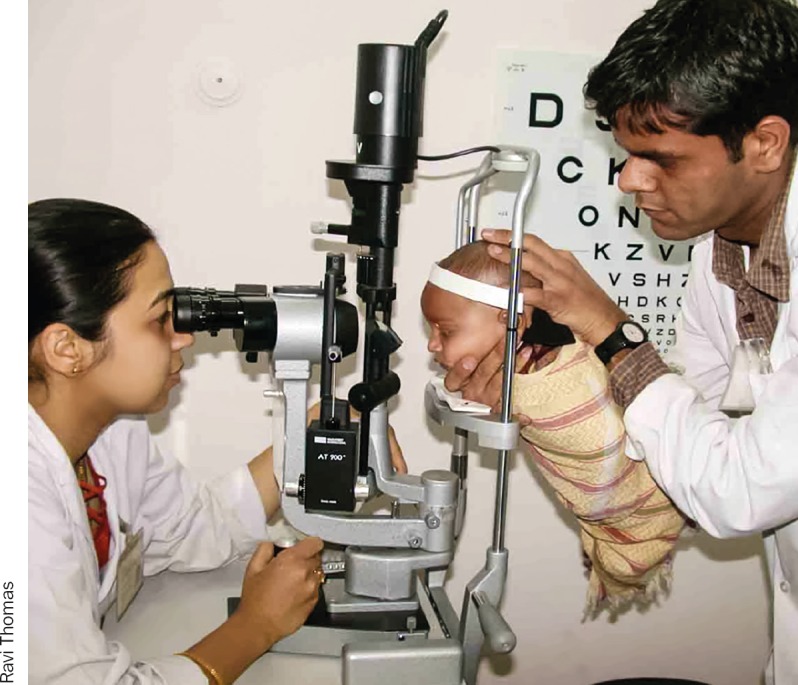
The ‘flying baby’ method for examining a baby at a slit lamp when there is no portable slit lamp available

**Figure 2.**
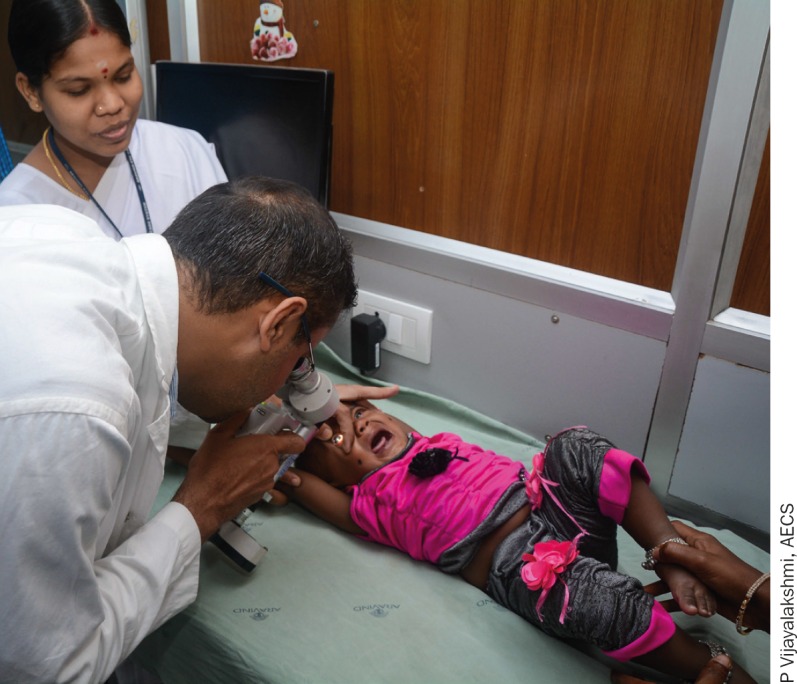
Using a portable slit lamp to examine a young child.

**Figure:**
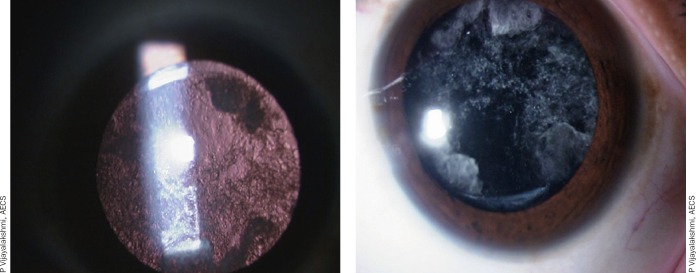
Paediatric cataract viewed using a slit lamp (left) and a diffuse light (right)

Routine examination on the first and second postoperative days should include the status of the wound, corneal clarity, anterior chamber reaction and depth, patency of the peripheral iridectomy (if performed), clarity of the visual axis, details of IOL placement and adequacy of pupil dilation. Parents should be asked if their child has shown signs of pain or discomfort.

Even young infants can be examined using a standard slit lamp, using the ‘flying baby’ method (Figure 1). A hand-held slit-lamp can be used if available (Figure 2). A hand-held light source, shone obliquely into the eye, can also be used to assess the anterior segment. The structures can be magnified using a 20- or 30-dioptre lens.

A minimum in-patient stay of two days is recommended for uncomplicated cases. The stay can be longer if there is intense postoperative inflammation or other complications and for those who may not come back for follow-up.

Two examples of routine topical medication (from day 1 after surgery) are given in the panel. Parents must be properly counselled so they understand how important it is to comply with medication, especially in the early postoperative period. Parents need to be shown how to instil the medication, which may entail showing parents how to swaddle their child and gently open the eye. Nursing staff need to be sure that parents can do this safely and reliably before the child is discharged.

## Control of inflammation

Systemic steroids are indicated if increased inflammation is anticipated (in cases of traumatic or complicated cataracts), or if there was severe inflammation following surgery on the first eye. A single dose of intravenous dexamethasone (4 mg per 25 kg of body weight) at the end of surgery helps to reduce inflammation. If severe inflammation persists after surgery, oral prednisolone can be used (0.5 mg to 1.0 mg per kg body weight) titrated according to the severity of inflammation and administered on a daily basis for the first 15 days, followed by the same dose on alternate days for the next 15 days.

An alternative is a single dose of subconjunctival or peribulbar triamcinolone 20–40 mg given at the end of surgery, especially when compliance is not guaranteed, or in younger children who cannot take oral medication. In many centres this is routine practice.

**Postoperative medication (topical)**

**Example 1 India**

**Non-IOL surgery**

- **Antibiotics and steroids:** For children under 12 months, use an antibiotic-steroid combination in ointment form (tobramycin and dexamethasone, or moxifloxacin and dexamethasone) 3 to 4 times a day, reducing over 6 weeks. For children over 12 months, give the same combination in eyedrop form according to the same schedule.
- **Dilation:** For children under 12 months, give atropine eye ointment once a day for 1 week, then twice a week for 3 weeks and finally once a week for 2 more weeks. For children over 12 months, use homatropine or cyclopentolate eyedrops according to the same schedule.

**IOL surgery**

- Give steroid eyedrops (predforte acetate 1%) 6 times a day, tapered over 6 weeks.
- Give antibiotic eyedrops (tobramycin or moxifloxacin) 4 times a day for 2 weeks, 3 times a day for the next 2 weeks and then twice a day for 2 more weeks.
- Dilating drops (homatropine eyedrops 1%) once a day for 2 weeks, and then every second day for 2 more weeks. Use ointment if the drops are too difficult to administer.

**Example 2 Kenya**

For both IOL and non-IOL surgery, the following regime is followed.

- **Topical steroid drops** (dexamethasone or prednisolone): initially 2-hourly for 2 weeks, then 4-hourly for a month, and finally tapered over another month if there is no evidence of inflammation. IOP monitoring is mandatory to check for steroid response.
- **Topical antibiotic drops** (chloramphenical, tobramymicin, quinololones): 4 times daily for 4 weeks.
- **Combined steroid and antibiotic preparations** can be used as an alternative, especially where drug availability, administration and compliance are an issue.
- **Ointments** can be used at night to enhance drug concentration and in those who are non-compliant with drops.
- **Atropine 1% drops:** once to twice daily for 2–4 weeks. For children younger than one year old, apply once daily or use the 0.5% preparation.

## On the day of discharge

The following should be checked:

- Visual acuity (if possible)
- Intraocular pressure (with a non-contact tonometer). It is important to avoid applying pressure on the eye as this can lead to an erroneous reading and can put pressure on the wound, leading to shallowing of the anterior chamber.
- Red reflex (using a direct ophthalmoscope)
- Posterior pole of the fundus (using an indirect ophthalmoscope).

After discharge, children are encouraged to wear dark glasses (sunglasses) for both protection and comfort.

## Initial follow-up

Children with complications should be reviewed weekly until improvement is noted.

The first follow-up visit for uncomplicated cases must be within 2–4 weeks after surgery. If possible, children should undergo refraction at this first postoperative visit; this minimises travelling for the parents and reduces the likelihood of missed follow-up appointments. Children undergoing cataract surgery (with or without IOL), should be dispensed spectacles within 2 weeks of cataract surgery. In older children who have undergone IOL surgery, the prescription of spectacles can be delayed until 4 weeks after surgery to allow the wound and refraction status to stabilise. (Where follow-up is uncertain, however, it is better to dispense spectacles on discharge). Remind parents about the importance of compliance with the prescribed eyedrops. Find out if they have any problems and support them to find solutions.

### Optical correction: non-IOL surgery

**Figure 3.**
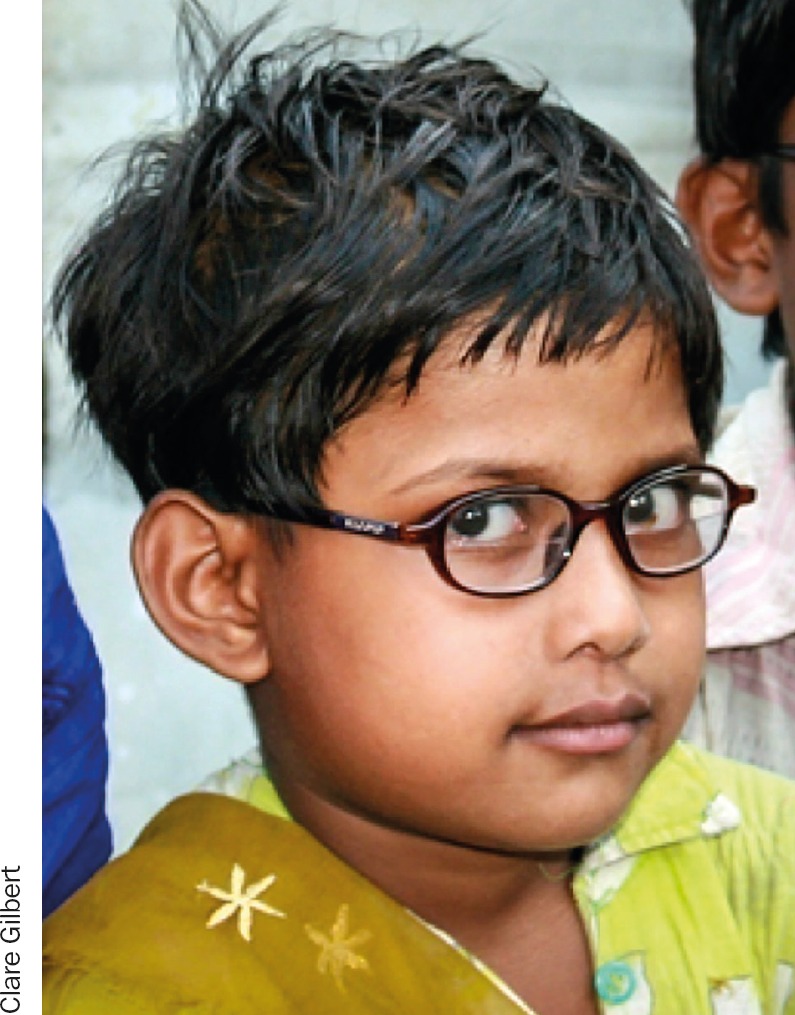
A Bangladeshi child wearing executive bifocal spectacles after bilateral cataract surgery.

Prescribe single lenses, focusing on near vision until the age of 18–36 months and bifocals after that. Contact lenses are another option. Children older than 3 years benefit from bifocals with a +2D add. A flat top D-shape or executive bifocal are preferred in children as they give a wider field of view and less distortion (Figure 3); however, they may not be readily available with the high plus lenses required by children with aphakia. Although progressive lenses give very good visual quality, they are expensive and not recommended for children as their spectacles need to be changed very frequently.

### Optical correction: IOL surgery

Any residual refractive error, especially astigmatism, should be corrected with an appropriate near vision addition, either in the form of bifocals or progressive lenses (depending upon the affordability) at the first postoperative visit. At each visit, compliance with spectacle wear should be discussed and any issues resolved. In cases of children with disabilities, this should be done with extra care, always encouraging the parents towards better compliance.

## Longer-term follow-up

Longer-term follow-up visits should take place every 3 months up to 2 years of age, every 6 months up to 5 years of age and thereafter yearly or as indicated until the child reaches maturity.

At each visit, the examination should include assessment of the visual acuity, refraction, and slit lamp examination for anterior segment details including IOL placement, pupil shape, clarity of the visual axis, any anterior chamber reaction, and measurement of intraocular pressure (using non-contact methods). Axial length measurement (especially in unilateral cataract or anisometropia) and fundus examination are also essential. Ocular motility and alignment should be assessed so that strabismus and/or amblyopia can be detected early.

Medication and advice (e.g patching) should be adjusted according to the findings. Extended medication should be given to those who may not return for follow-up due to travel logistics or financial constraints. Many families need reminders and special help (reimbursement) for follow-up. It is useful to have one dedicated person in the team to monitor this.

